# Graphene at Liquid Copper Catalysts: Atomic‐Scale Agreement of Experimental and First‐Principles Adsorption Height

**DOI:** 10.1002/advs.202204684

**Published:** 2022-11-09

**Authors:** Hao Gao, Valentina Belova, Francesco La Porta, Juan Santiago Cingolani, Mie Andersen, Mehdi Saedi, Oleg V. Konovalov, Maciej Jankowski, Hendrik H. Heenen, Irene M. N. Groot, Gilles Renaud, Karsten Reuter

**Affiliations:** ^1^ Fritz‐Haber‐Institut der Max‐Planck‐Gesellschaft Faradayweg 4–6 14195 Berlin Germany; ^2^ The European Synchrotron‐ ESRF 71 Avenue des Martyrs, CS 40220 Grenoble Cedex 9 38043 France; ^3^ Chair for Theoretical Chemistry and Catalysis Research Center Technische Universität München Lichtenbergstraße 4 85747 Garching Germany; ^4^ Aarhus Institute of Advanced Studies & Center for Interstellar Catalysis Department of Physics and Astronomy Aarhus University Aarhus C DK‐8000 Denmark; ^5^ Leiden Institute of Chemistry Leiden University P.O. Box 9502 RA Leiden 2300 The Netherlands; ^6^ Université Grenoble Alpes CEA, IRIG/MEM/NRS Grenoble 38000 France

**Keywords:** graphene, liquid metal catalyst, machine learning, X‐ray reflectivity

## Abstract

Liquid metal catalysts have recently attracted attention for synthesizing high‐quality 2D materials facilitated via the catalysts’ perfectly smooth surface. However, the microscopic catalytic processes occurring at the surface are still largely unclear because liquid metals escape the accessibility of traditional experimental and computational surface science approaches. Hence, numerous controversies are found regarding different applications, with graphene (Gr) growth on liquid copper (Cu) as a prominent prototype. In this work, novel in situ and in silico techniques are employed to achieve an atomic‐level characterization of the graphene adsorption height above liquid Cu, reaching quantitative agreement within 0.1 Å between experiment and theory. The results are obtained via in situ synchrotron X‐ray reflectivity (XRR) measurements over wide‐range q‐vectors and large‐scale molecular dynamics simulations based on efficient machine‐learning (ML) potentials trained to first‐principles density functional theory (DFT) data. The computational insight is demonstrated to be robust against inherent DFT errors and reveals the nature of graphene binding to be highly comparable at liquid Cu and solid Cu(111). Transporting the predictive first‐principles quality via ML potentials to the scales required for liquid metal catalysis thus provides a powerful approach to reach microscopic understanding, analogous to the established computational approaches for catalysis at solid surfaces.

## Graphical abstract

A quantitative atomic‐scale understanding of liquid metal catalysts is important for the high‐quality synthesis of graphene. Using machine‐learning potentials trained to density functional theory data, the authors achieve sub‐angstrom agreement to the experimentally determined graphene adsorption height on liquid Cu. This work draws a path for the use of reliable potentials as a multiscale modeling technique to explore unchartered problems.

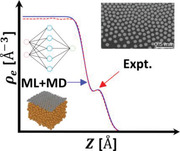

## 1 Introduction

The recent discovery of rapid and high‐quality synthesis of graphene (Gr) at liquid copper (Cu) catalysts was an important finding toward large‐scale commercialization.^[^ ^1^, ^2^ ^]^ At present though, there is predominantly only phenomenological mechanistic understanding of what may lead to the improved Gr quality as compared to the more traditional synthesis on solid Cu catalysts, which typically employs highly comparable growth conditions just at ≈100 K lower temperature. Previously suggested aspects include the absence of extended defects, generally higher mobility of atoms, clusters and flakes, or an increased carbon dissolution at the liquid Cu surface.^[^ ^3^, ^4^, ^5^, ^6^, ^7^, ^8^, ^9^ ^]^ The precise role of these effects is not quantitatively established though. Therefore, detailed atomic‐scale investigations are highly desired to further our understanding, carve out the governing factors, and then employ this for a rational improvement of the growth process—a strategy that has long been successfully exercised for solid catalysts.

Unfortunately, such atomic‐scale investigations are severely challenged by growth at liquid catalysts. In situ measurements not only have to deal with the reactive gas‐phase conditions but also with the elevated temperatures, for example, 1400 K in the case of Cu.^[^ ^10^, ^11^, ^12^, ^13^ ^]^ On the modeling and simulation side, predictive‐quality first‐principles calculations are the major contemporary workhorse for atomic‐scale mechanistic studies at solid metal catalysts.^[^ ^14^, ^15^ ^]^ Yet, they impose excessive computational costs for liquid metal surfaces. Capturing the increased dynamics and reduced symmetries of a liquid metal surface requires sampling over length and time scales far beyond the often statically performed calculations at rigid active site motifs in solid catalyst studies.^[^ ^16^ ^]^ Furthermore, already the latter calculations are at present only practically feasible within semi‐local density functional theory (DFT). To reach a high predictive quality on the basis of the involved approximate DFT functionals, much knowledge has been gathered about their applicability to different classes of reactions at solid catalysts. However, how much of this knowledge can be transferred to the liquid state is presently unclear. In this situation, not even the choice of the DFTfunctional on which one would base first‐principles simulations of growth at a liquid catalyst could be well motivated.

Fortunately, much progress has recently been achieved on both experimental and modeling sides. Improved operando measurement setups and subsequent data analyses provide more and increasingly accurate atomic‐scale observables. Here, we illustrate this by now achievable wide‐range q‐vector in situ X‐ray reflectivity (XRR) measurements in a tailored reactor setup^[^ ^7^, ^11^, ^17^ ^]^ that provide the adsorption height of a Gr monolayer above the liquid Cu catalyst (the so‐called “gap”) at unprecedented accuracy. On the modeling side, the advent of machine‐learning (ML) potentials constitutes a breakthrough.^[^ ^18^, ^19^, ^20^ ^]^ Trained with first‐principles data, these ML surrogate models enable atomistic simulations and sampling comparable to classical force fields while reliably propagating the first‐principles predictive quality even to the reactive surface chemistry characteristic for growth at liquid catalysts. Here, we exploit this novel methodology to quantitatively determine the “gap” and compare it to the experimental reference. Further, we also exploit the low computational demand and high data efficiency of the approach to train ML surrogate models with data calculated by different DFT functionals. This provides an unprecedented means to directly assess how much the functional uncertainty propagates to the “gap” and other literature observables for liquid Cu. Intriguingly we find only a low sensitivity and, furthermore, a clear correlation to easily accessible crystalline descriptors. This establishes firm guidance for choosing DFT functionals in future first‐principles ML surrogate‐based mechanistic studies for catalysis at liquid metal surfaces.

## 2 Experimental Section

### 2.1 Graphene Synthesis

**Figure 1:**
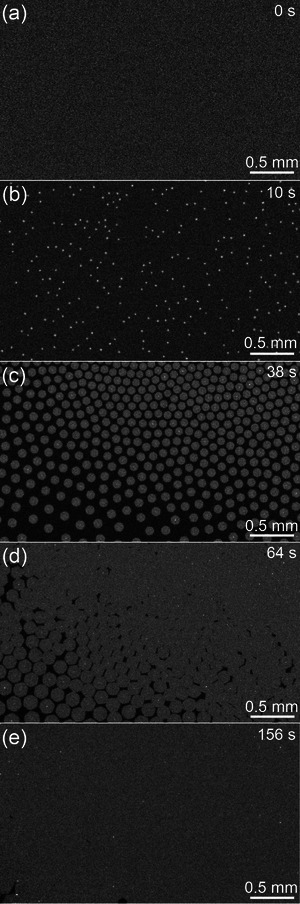
In situ optical microscopy images of different graphene growth stages: a) liquid Cu before growth; b) shortly after nucleation; c) flakes self‐assembly; d) flakes merging; and e) closing of monolayer sheet.

Monolayer Gr was synthesized on liquid Cu at 1370 K via a previously established chemical vapor deposition (CVD) growth protocol using methane as a precursor in a reactor fitted with in situ characterization techniques that allow for the monitoring of the growth process.^[^ ^7^, ^11^ ^]^ With further details provided in the Supporting Information, the in situ optical microscopy results in **Figure** **1**a,b demonstrate that Gr nucleation was initiated after a “pulsed” high concentration methane burst at time 0 s, followed by continuous flake growth at lower methane concentration (*p*_CH4_/*p*_H2_ = 0.0127)^[^ ^7^ ^]^ as shown in Figure 1c,d. In the final growth stage, the large macroscopic flakes merge into a single‐layer Gr sheet, as shown in Figure 1e. The final result was a polycrystalline monolayer Gr composed of single‐crystal domains with a size of a few hundred micrometers on average. More details on the growth procedures can be found in Ref. [7].

### 2.2 X‐Ray Reflectivity Measurements

**Figure 2:**
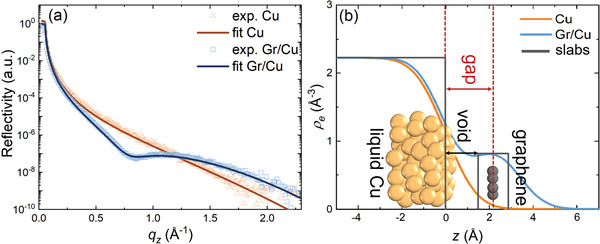
a) In situ XRR scans of liquid Cu (orange) and liquid Cu covered with a graphene monolayer (blue) together with the corresponding fit; and b) electron density profiles obtained from the experimental XRR curves. The grey rectangles present the model slab layers with zero roughness.

The adsorption height of the Gr monolayer above the liquid Cu surface (“gap”) was determined by in situ XRR at the ID10 beamline of the ESRF synchrotron (Grenoble, France). The XRR scans were treated according to the method described recently in Ref. [17] which not only accounts for the effect of the liquid surface curvature but also takes advantage of it. This, together with an improved reactor setup (e.g., larger sample surface) and measurement methodology, allowed the authors to extract the normalized reflectivity profiles up to an unprecedented maximum value of the momentum transfer perpendicular to the surface, *q*_z_. **Figure** **2**a shows the resulting profiles before and after Gr growth (corresponding to Figures 1a and 1e, respectively). Employing the Parratt formalism,^[^ ^21^ ^]^ the reflectivity curves were fitted using a slab model consisting of one (in case of bare Cu) or three (in case of Cu covered with Gr) layers: signifying a Cu substrate, a void, and a carbon monolayer. With all details provided in the Supporting Information and Ref. [17], this fit yields the lateral average electron density profile against the surface depicted in Figure 2b. From this profile, the “gap” distance was determined as the distance between the inflection point of the Cu density and the center of the Gr slab/layer, as indicated by the dashed lines in Figure 2b. The advantage of using this Cu inflection point is a “gap” distance that does not depend on the Cu roughness. Minor deviations of the below‐quoted value compared to previous reports^[^ ^7^, ^17^ ^]^ can be explained by the much shorter *q*_z_ range available for fitting (between 0.4–1.6 Å^−1^) in these preceding studies.

### 2.3 Machine‐Learning Potential

Moment Tensor Potentials (MTP)^[^ ^22^, ^23^ ^]^ had been trained with DFT reference data to establish a numerically efficient ML surrogate model. The small number of hyperparameters involved in MTPs allows for a simple out‐of‐the‐box application further described in the Supporting Information. The set of training and test structures (see Table S1, Supporting Information) was initially comprised of (strained) crystalline and liquid Cu bulk cells, (strained) free‐standing periodic Gr sheets, and a finite Gr flake on a large liquid Cu slab. Interfacial information was subsequently provided by adding structures of a graphene sheet at various heights above a snapshot of a liquid Cu surface to the training set. All the liquid Cu structures were thereby taken as snapshots from preceding ab initio molecular dynamics work on this system.^[^ ^16^ ^]^ The DFT supercell calculations were performed with the full‐potential, all‐electron package FHI‐aims,^[^ ^24^ ^]^ with all computational details provided in the Supporting Information. The entire set for training and test was computed with the Perdew–Burke–Ernzerhof (PBE) functional,^[^ ^25^ ^]^ once adding Tkatchenko–Scheffler (TS)^[^ ^26^ ^]^ dispersion and once adding many body dispersion (MBD)^[^ ^27^ ^]^ corrections, as well as with the regularized strongly constrained and appropriately normed (rSCAN) functional.^[^ ^28^ ^]^ This leads to four trained MTPs (MTP@PBE, MTP@PBE+TS, MTP@PBE+MBD, MTP@rSCAN) that all exhibit a high accuracy and transferability as expressed by root‐mean‐squared errors (RMSE) below 2 meV/atom (6.3 meV/atom) in energies and below 55 meV Å^−1^ (96 meV Å^−1^) in the forces over the training (and test) set (see Figure S1 and Table S2, Supporting Information). The authors note that the difference in RMSE between training and test sets indicates slight overfitting, yet without compromising accuracy.

### 2.4 Molecular Dynamics Simulations

**Figure 3:**
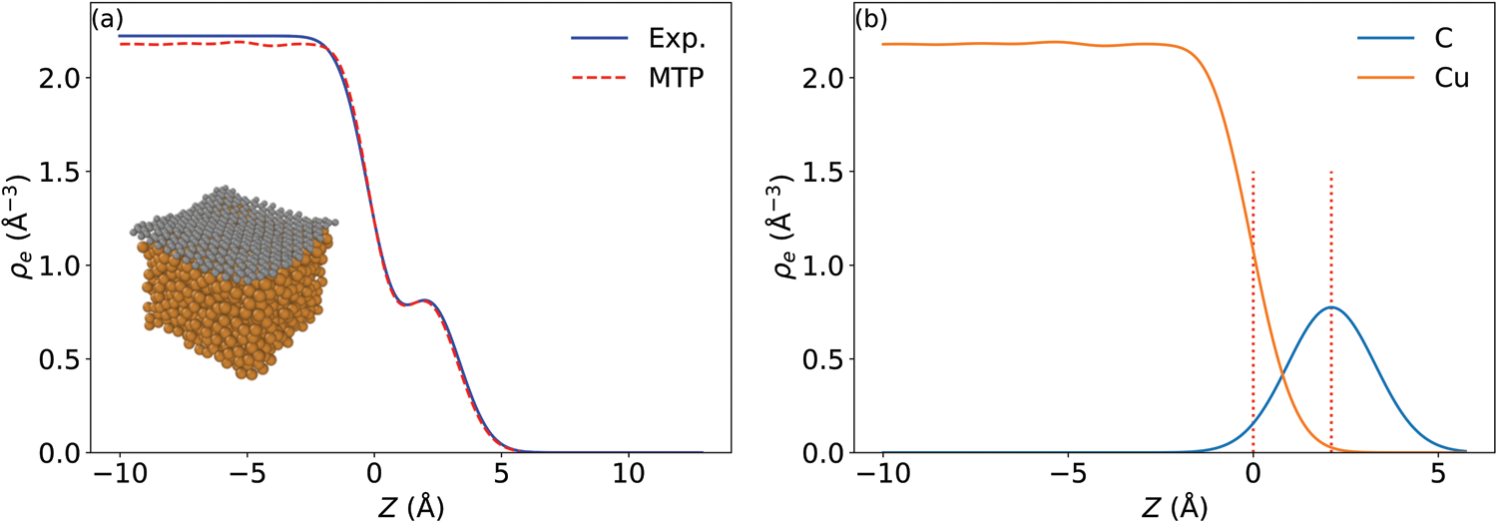
a) Electron density profiles from experiment and computed by MTP@PBE+MBD‐based MD at 1370 K (using a Gaussian width *σ*_Gauss_ = 0.9 Å for Cu and 1.1 Å for C, see Supporting Information). b) The corresponding species‐resolved computed electron density profiles of Cu and C. Vertical dashed lines denote the Cu density inflection point (chosen as zero reference) and the peak of the C Profile (denoting the Gr position). The “gap” is derived as the distance between these two heights.

The high efficiency of the trained MTPs allows for extensive sampling via large‐scale classical molecular dynamics (MD) simulations using the atomic simulation environment (ASE)^[^ ^29^ ^]^ and the Large‐scale Atomic/Molecular Massively Parallel Simulator (LAMMPS) package.^[^ ^30^ ^]^ Bulk liquid Cu simulations were conducted in a supercell with 864 atoms in the isothermal‐isobaric ensemble at 1356 K to provide the bulk liquid density. Surface liquid Cu canonical ensemble simulations involve a 1589 Cu atom slab and yield the surface tension. Adding a 364 C atom Gr sheet, as shown in **Figure** **3**a, gives access to the “gap” on liquid Cu (Gr—Cu(l)). For Gr on solid Cu(111) (Gr—Cu(111)), a large simulation cell composed of a slab with 11664 Cu atoms (corresponding to a centered rectangular c(27 × 27√3) Cu(111) surface unit‐cell and eight atomic layers) covered with a Gr sheet containing 3136 C atoms was used to compute the “gap” on solid Cu. Here, the lattice constant of Gr was adjusted according to the relaxed lattice of Cu at 0 K. For MTP@PBE+MBD, the lattice mismatch in this large simulation cell leads to a residual compression of only 0.1% of Gr at 0 K. Two smaller models of Gr—Cu(111) in rectangular c(13 × 7√3) and c(24 × 24√3) Cu(111) surfaces with strains of +3.6% and −0.5% at 0 K, respectively, were further compared (see details in the Supporting Information).

For all investigated configurations and MTPs, MD simulations were propagated for 1 ns, which yielded converged averaged quantities as explicitly demonstrated for the “gap” in Figure S3, Supporting Information. Further, energetic convergence was also ensured, as shown in Figure S6, Supporting Information, where most of the drift in total energy was less than 0.5 meV/(ns∙atom). To quantitatively evaluate the liquid “gap”, a Gaussian‐shaped spherical electron density of width *σ*_GAUSS_ was assigned to each atomic position to subsequently transform the trajectory data into a time and laterally averaged electron density profile along the surface normal that was directly comparable to the experimentally obtained density shown in Figure 2b. The Gr—Cu “gap” was then determined by the analog Cu inflection‐point measure used in the experimental analysis. As detailed in Figure S2, Supporting Information, the thus determined value was essentially insensitive to the Gaussian width *σ*_GAUSS_ employed in the construction of the electron density. In this work, this definition of gap is called the inflection‐point “gap” and is adhered to for all “gap” value evaluations. Furthermore, since the surface atoms’ positions can be well defined for a solid slab, a direct method for the Gr—Cu “gap” was also evaluated in the Supporting Information. This value was usually determined for solid substrates in experiments.^[^ ^31^ ^]^ As evaluated in the simulations (see Supporting Information), between these two “gap” definitions can be translated by a constant offset of *δD* = 1 Å to compare “gaps” on solid and liquid Cu. All the details on the evaluation of properties and their uncertainties are shown in the Supporting Information.

## 3 Results and Discussion

### 3.1 Graphene Adsorption Height

Figure 3a compares the experimental and computed electron density profiles of the liquid Cu surface covered with a Gr sheet at the same temperature of 1370 K. Specifically, we compare the computed density obtained with the MTP@PBE+MBD potential, that is, the MTP trained on the DFT data calculated with the PBE functional and MBD dispersion correction. The two curves are in essentially perfect quantitative agreement. The slightly higher bulk plateau of the experimental curve reflects the slightly underestimated liquid Cu density of DFT PBE+MBD (see below). On the bulk plateau, the computed density profile exhibits some minor oscillations near the Cu surface region (*z* ≈ −3–4 Å), which indicate some residual surface layering (compared to the atomic density profile in Figure S5c, Supporting Information). This would not be untypical for liquid metals, but is not detected in the experimental spectra. We stress that the present MD simulations cannot capture macroscopic effects like capillary waves, which, however, are detectable in the experiment.^[^ ^10^ ^]^ We correspondingly believe that a resulting averaging effect of capillary waves may explain the absence of such oscillations in the experimental profile.

The agreement between the measured and computed curve particularly extends over the hump in the decaying electron density profile. As is apparent from the species‐resolved computed electron density profile shown in Figure 3b, this hump denotes the position of the Gr layer. Therefore, the excellent agreement of the curves in this region translates to a quantitative agreement of the derived Gr adsorption height. The experimental “gap” is determined to be 2.2 ± 0.1 Å, while the MTP@PBE+MBD “gap” comes out as 2.119 (±0.003) Å. The latter value should be compared to the 2.86 and 2.89 Å reported previously^[^ ^7^, ^16^ ^]^ from extensive MD simulations based on the variable‐charge reactive force field COMB3,^[^ ^32^, ^33^ ^]^ which would, by all means, be deemed an adequate state‐of‐the‐art force field for this system. The COMB3 value was furthermore supported by preliminary, short‐trajectory ab initio MD simulations based on PBE together with TS dispersion correction.^[^ ^16^ ^]^ It is thus only the combination of both extensive sampling and first‐principles accuracy enabled by the ML surrogate model that resolves the discrepancy and now firmly establishes a reference value for the adsorption height of Gr above liquid Cu backed by both experiment and theory.

### 3.2 Liquid Versus Solid Cu

With this reference established for the liquid metal surface, we can now compare the “gap” reported at both liquid and solid Cu. The inflection‐point gap on Cu(111) was previously measured at room temperature via ex situ total‐reflection high‐energy positron diffraction (TRHEPD) and atomic force microscopy (AFM) as 2.34 ± 0.06^[^ ^31^ ^]^ and 2.0 ± 0.2 Å,^[^ ^34^ ^]^ respectively (corresponding to a direct “gap” of 3.34 ± 0.06 and 3.0 ± 0.2 Å). Unfortunately, the large scatter between these two experimental values and their inherent uncertainty renders it impossible to draw any conclusions on a difference between the “gap” at solid and liquid Cu. Such a difference could be potentially induced by a more considerable dynamic buckling of Gr on liquid Cu that gives rise to a somewhat stronger sp^3^ activation of Gr and a concomitant chemical interaction in addition to the dispersive interaction known to dominate the binding of Gr on solid Cu(111). To this end, we resort to an MTP@PBE+MBD based computation, which we conduct in the gigantic minimal‐strain c(27 × 27√3) simulation cell for reasons apparent below. The resulting value for the “gap” on Cu(111) at 300 K is 2.259 (±0.0002) Å (see **Table** **1** and Figure S2, Supporting Information), that is, ≈0.14 Å larger than the “gap” computed at liquid Cu at 1370 K.

**Table 1:** Inflection‐point gaps as computed by ML potentials trained with DFT reference data at different functionals and as measured by experiment

	MTP@rSCAN	MTP@PBE	MTP@PBE+TS	MTP@PBE+MBD	Exp. (this work)
Gr—Cu(l) gap at 1370 K [Å]	2.353 ± 0.009	2.956 ± 0.014	1.971 ± 0.003	2.119 ± 0.003	2.2 ± 0.1

**Figure 4:**
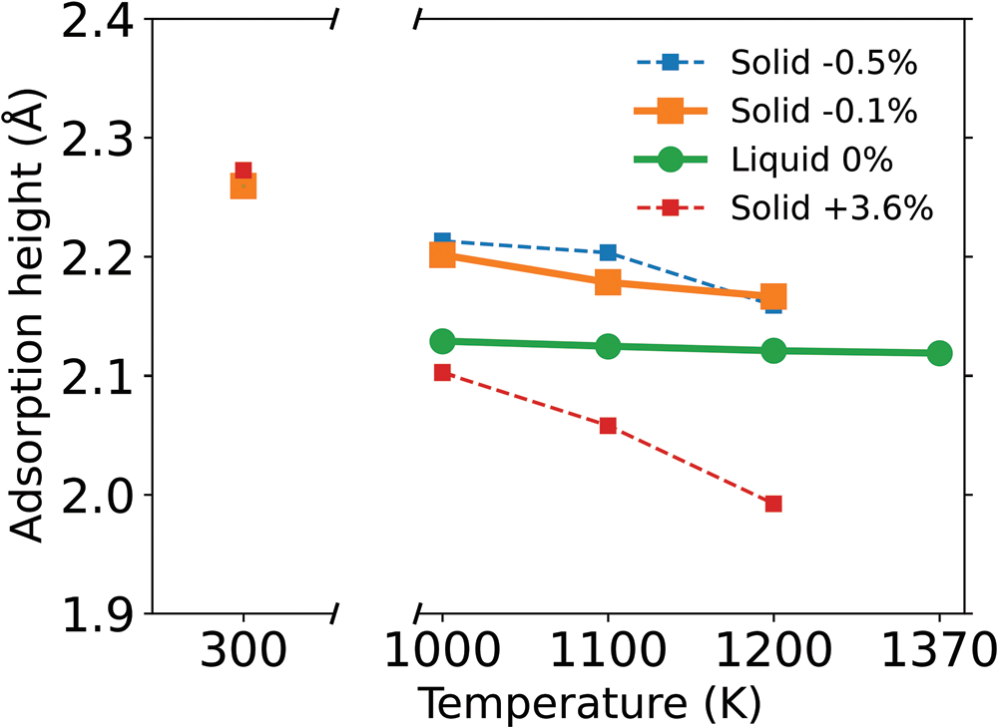
The inflection‐point gap of Gr on liquid Cu and solid Cu(111) (with different Gr‐sheet strains as indicated in the legend) against simulation temperature for the MTP@PBE+MBD potential. The −0.1% data points correspond to the gigantic c(27 × 27√3) simulation cell employed for the quantitative “gap” comparison with the experiment.

While this difference could indeed reflect an additional chemical interaction at the liquid Cu catalyst, it could also merely be the result of anharmonic vibrations at the differing temperatures (300 K versus 1370 K). Therefore, we proceed to compute the Gr gap on solid and liquid Cu at the same elevated, but a slightly reduced temperature of 1200 K compared to the Cu melting point, respectively. This temperature is chosen since it is close to the estimated melting point of PBE‐DFT,^[^ ^35^ ^]^ and we here exploit that on MD timescales of 1 ns, the melting of the solid or freezing of the liquid does not yet set in for the employed simulation cells (compare Figure S6, Supporting Information) and the simulation of Gr—Cu(l) corresponds to a supercooled state. These simulations yield inflection‐point “gaps” of 2.121 ± 0.002 and 2.165 ± 0.004 Å, for liquid and solid Cu, respectively (see also **Figure** **4**). They are thus virtually identical at the same temperature (Δ = 0.044 Å), which suggests an essentially identical nature of the bonding at both solid and liquid Cu that is predominantly dispersive. In addition, this interpretation is corroborated by highly similar adsorption energies of −48 meV/atom on liquid versus −40 meV/atom on solid Cu(111) at 1200 K, as well as highly similar d‐band center positions as a ruling descriptor for chemisorptive interactions.^[^ ^36^ ^]^

**Figure 5:**
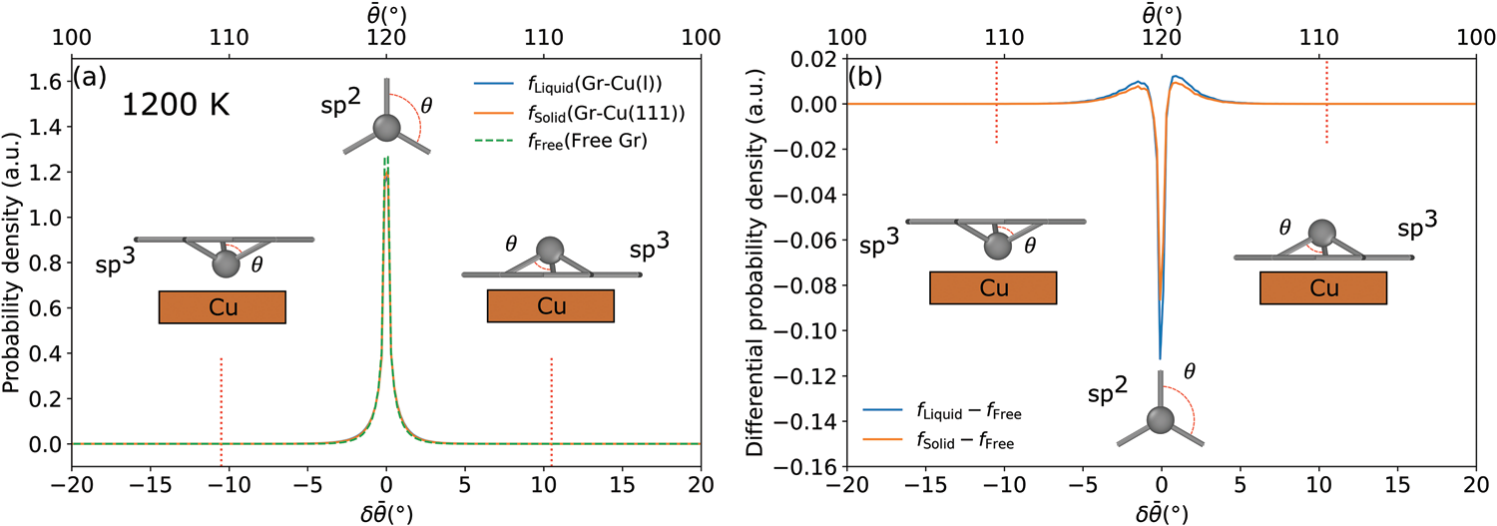
a) Signed average differential bond angles ($\delta \bar{\theta }$, lower *x*‐axis) and the corresponding average angle ($\bar{\theta }$ upper *x*‐axis) inside the Gr layer with and without liquid/solid Cu slab at 1200 K. The zero reference is planar sp^2^‐bonded Gr. The red dotted lines indicate the typical bond angle in sp^3^ hybridization. Negative angles indicate an orientation below the Gr sheet and positive values above it. The typical neighboring environments with negative and positive $\delta \bar{\theta }$ are shown by insets. Note that the probability densities show only marginal differences for free Gr, as well as Gr on liquid (Gr—Cu(l)) and solid Cu (Gr—Cu(111)). b) The difference between the distributions of $\delta \bar{\theta }$ and $\bar{\theta }$ with respect to free‐standing Gr.

To further rule out a significant sp^3^‐type Gr activation at liquid Cu, we characterize the bonded state of Gr on liquid and solid Cu(111) by analyzing the Gr geometry during the MD simulations at 1200 K. Specifically, we evaluate the distribution of the average differential bond angles of coordinating C atoms $\delta \bar{\theta }$ for all the C atoms inside Gr for free‐standing Gr and Gr adsorbed on solid and liquid Cu. As a prior for the local coordination environment, values of $\delta \bar{\theta }$ of 0° and ±10.5° represent sp^2^‐ or sp^3^‐hybridized C, respectively. Negative values indicate a C “bending” toward the Cu surface, and positive values a C “bending” away from the surface. As shown in **Figure** **5** (and Figure S4, Supporting Information, for liquid Cu at the even more elevated temperature of 1370 K), the angle distributions are essentially identical for Gr at liquid and solid Cu. This confirms the above conclusion of an absence of additional sp^3^‐activation at the liquid metal surface. In fact, there is no significant sp^3^‐type hybridization at either surface, as clearly revealed from the absence of any peak at *δθ* = ±10.5°. Instead, in particular the difference to free‐standing Gr shown in Figure 5b demonstrates that the primary effect of the surface (regardless of whether solid or liquid) at this elevated temperature is to slightly modify the intrinsic longer‐range rippling of Gr as reflected by the additional broadening of the angle distribution ≈*δθ* = 0°.

We note that this quantitative comparison and concomitant conclusions are only enabled by the use of the gigantic c(27 × 27√3) minimum‐strain Cu(111) simulation cell. Figure 4 shows the temperature dependence of the adsorption height of Gr on solid and liquid Cu, also including data generated in models with smaller Cu(111) cells with a concomitantly increased Gr strain. In particular for the smallest such cell with a strain of +3.6%, a strong temperature dependence of the “gap” is obtained. In contrast, the liquid Cu “gap” shows almost no temperature dependence with “gaps” varying by Δ = 0.008 Å in the temperature range 1000 to 1200 K. The latter invariance further supports our direct comparison to the experimental data, even in light of the uncertainty with regards to the computational melting temperature of Cu at the PBE+MBD level. We attribute the overall decrease of the computed “gap” at solid Cu(111) with increasing temperature to the thermal expansion of the Gr lattice in the extended periodic cell and a thus resulting additionally induced small strain. The stronger this strain, the stronger the temperature dependence. The small difference in this temperature dependence obtained Figure 4 in for the solid Cu(111) computed in the c(24 × 24√3) cell model with −0.5% strain and the gigantic c(27 × 27√3) cell model with −0.1% strain suggests that the residual strain in the latter cell has no longer any significant effect on the deduced “gap”. At least, the remaining variations cannot explain the small difference in the computed solid and liquid gap at elevated temperatures of the order of Δ = 0.05 Å apparent in Figure 5. While we cannot exclude that this difference could indeed result from slightly different interactions, we emphasize that it may also result (at least partially) from sampling restrictions of the liquid state in the employed finite supercell. Even if the latter was the case though, its effect on the computed “gap” would thus be smaller than Δ = 0.05 Å, showing that we indeed reached a quantitative agreement between the computed and measured “gap” at liquid Cu within the experimental uncertainty.

### 3.3 DFT Functional Uncertainty

Unfortunately, the agreement between measured and computed “gap” does not yet take the most prominent systematic error of DFT into account, which arises from the employed approximate DFT exchange and correlation functional. At the present level of semi‐local functionals together with an additional dispersion correction, this specifically translates to the choice of the prior and the choice of the latter, as well as of the combination of both. As stated initially, little information is hitherto available concerning the suitability of prevalent functionals and dispersion corrections for liquid metal catalysts. To this end, we evaluate the sensitivity of the determined “gap” of Gr on liquid Cu and other liquid metal properties by training MTP potentials with DFT data computed with different functionals and dispersion corrections. This way, the ML surrogate models establish a generally applicable avenue to quality control of DFT by enabling the simulation of experimental observables that would not be feasible at the direct DFT level.

We fit three more MTPs at the same accuracy level (i.e., same MTP hyperparameters, cf. Supporting Information) and based on exactly the same training set as used before, the only difference being that this training data is now computed with the pure PBE and rSCAN functional, as well as at the PBE+TS dispersion‐corrected level. In the following, the newly trained ML potentials are denoted as MTP@PBE, MTP@PBE+TS and MTP@rSCAN, and we expect them to each be equally representative of the DFT functional used for their training data. On the one hand, the resulting series thus allows to compare two popular semi‐local functionals (PBE and rSCAN) which are known to under‐ and overbind Cu in the solid state (see below), respectively. On the other hand, it allows for an evaluation of the influence of varying dispersion corrections, ranging from PBE with no dispersion, over PBE+TS which includes pairwise‐additive dispersive Cu—C and C—C interactions, to PBE+MBD which includes many‐body dispersive interactions among all species.^[^ ^27^, ^37^ ^]^

**Figure 6:**
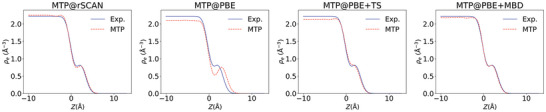
Electron density profiles as in Figure 3a, but for the different ML potentials, see text.

As apparent from Table 1, the different DFT levels yield quite a range of “gap” values, resulting in the concomitant varying degree of agreement with the measured electron density profiles from the experiment seen in **Figure** **6**. Intriguingly though, the obtained trend closely follows the expectations derived from the performance of the different levels of theory for bulk solid Cu and for Gr on Cu(111). The known underbinding of the pure PBE functional and complete absence of dispersive interactions at this level of theory yields a grossly overestimated “gap” value of almost 3 Å. The known overbinding of rSCAN reduces this error significantly, but the “gap” still comes out too large. This illustrates that the employed short‐range functional can effectively compensate missing dispersive contributions to some extent. Correspondingly, the combination of short‐range functional and dispersion correction needs to be well balanced. At solid Cu(111), the PBE+TS combination is known to overbind between Cu and C,^[^ ^38^, ^39^ ^]^ and we obtain exactly the same for Gr at liquid Cu. The “gap” becomes too small. It is, therefore, the reduced many‐body dispersive interactions at the PBE+MBD^[^ ^27^, ^40^, ^41^ ^]^ level that achieve the right balance—exactly as expected from insight gained through adsorption calculations of (hydro)carbons at solid Cu.^[^ ^42^, ^43^ ^]^

**Figure 7:**
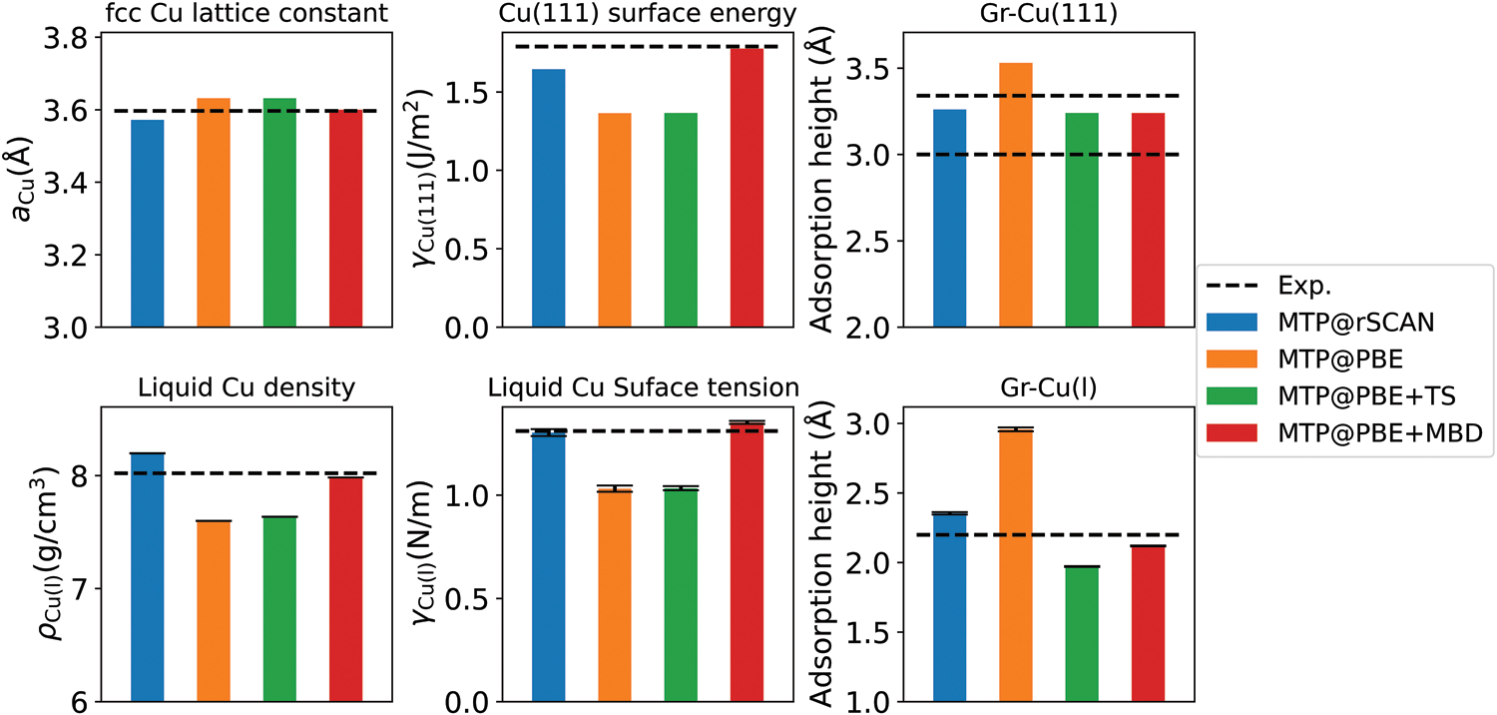
Correlation in the performance of different levels of theory on analog observables of solid (upper panels) and liquid Cu (lower panels): (left panels) *T* = 0 K Cu fcc lattice constant (*a*_Cu_) versus liquid Cu density (*ρ*_Cu(l)_) at *T* = 1356 K (experimental melting temperature of Cu), (middle panels) *T* = 0 K Cu(111) surface energy (*γ*_Cu(111)_ versus *T* = 1370 K liquid Cu tension (*γ*_Cu(l)_, and (right panels) *T* = 0 K Gr—Cu(111) “gap” versus the *T* = 1370 K (experimental temperature in this work) Gr—Cu(l) “gap”. The computational uncertainties are shown by error bars. Experimental reference data are taken from Refs. [31, 34, 44, 45, 46, 47] and this work, see text. For the Gr—Cu(111) “gap” two experimental references are shown.

This suggests that much of the knowledge gathered in calculations on bulk solids and solid surfaces can be transferred to liquid metal catalysis. **Figure** **7** compiles more data for bulk Cu and clean Cu(111) that supports this interpretation. For the latter, we retrained the varying MTPs exclusively on the subset of pure Cu training structures, that is, we disregarded all Gr—Cu structures to eliminate any residual influence of the latter on the learned Cu—Cu potential, cf. Supporting Information for details. Figure 7 juxtaposes analog computed observables of the solid and liquid, and each time compares them to the experiment: the *T* = 0 K Cu fcc lattice constant versus the liquid Cu density at *T* = 1356 K (exp. data from Refs. [44, 45]), the *T* = 0 K Cu(111) surface energy versus the *T* = 1370 K liquid Cu tension (exp. data from Refs. [46, 47]), as well as the *T* = 0 K Gr—Cu(111) “gap” versus the *T* = 1370 K Gr—Cu(l) “gap” (exp. data from Refs. [31, 34] and this work). In all three cases, we obtain exactly the same trends over the four levels of theory between solid and liquid state properties. Intriguingly, this even extends to anomalies previously noted for the solid state:^[^ ^48^ ^]^ For the bulk structural properties, Cu lattice constant and liquid density, pure rSCAN overbinds, and pure PBE underbinds. Since we do not let the +TS correction act on Cu—Cu interactions, this level of dispersion correction does not change anything here, while the MBD correction to PBE leads to an intermediate result. In contrast, for the energetic Cu surface properties, surface energy and surface tension, the MBD correction even overshoots rSCAN. We note, that we also find analog but less pronounced trends for the Gr lattice constant which illustrates the C—C interactions as summarized in Figure S7, Supporting Information. While the MTP ML‐surrogate models allow for quantitatively assessing the performance of different approximate exchange and correlation treatments for the calculation of liquid metal catalysts, it thus seems that knowledge of the performance of the respective solid metal catalyst provides already good guidance on their suitability. In this respect, this increases the confidence in the obtained PBE+MBD “gap” at liquid Cu, as there is ample experience from calculations of hydrocarbons at solid Cu that demonstrates the accuracy of this specific level of theory.^[^ ^42^ ^]^

## 4 Conclusion

Synthesis of 2D materials at liquid metal catalysts is a promising route to the large‐scale production of these unique materials with very high quality. In particular, atomic‐scale insight into why the growth at liquid surfaces excels the growth at solid surfaces could be critical for the further optimization of the process. To this end, we have established first‐principles trained ML‐potentials as a powerful approach that reliably transfers the predictive quality to the extensive sampling required to capture the liquid catalyst state in molecular simulations. Providing a firm experimental reference through the latest advanced in situ XRR measurements, we could show that such ML‐potential‐based simulations can provide the height of graphene above a catalytic liquid Cu surface with sub‐angstrom precision. The ability to predict quantitatively such a core quantity for 2D materials growth is a prerequisite to future detailed mechanistic investigations. Leveraging all advantages of these MLIPs, enhanced sampling methods could eventually be employed to study elementary reaction steps of the growth process and give access to their accurate free energy barriers.

The quality assertion notably extends to the systematic error due to the employed approximate DFT functional and dispersion correction. The possibility to train ML‐potentials with DFT data computed at different levels of theory and then efficiently compute liquid state properties allows us to analyze the respective suitability in an analog way as traditionally done through static direct DFT calculations in solid‐state catalysis. For Gr on liquid Cu, such analysis showed a strong correlation to the performance of short‐range functionals and their combination with various dispersion corrections known from Gr on solid Cu. If these correlations prevail for other systems, future DFT‐based studies of synthesis of 2D materials can rely their choice of a functional either on descriptors which are computationally much easier to obtain on solid surfaces, or the existing community knowledge built up over decades of work on solid‐state catalysts.

## Conflict of Interest

The authors declare no conflict of interest.

## Supporting information

Supporting Information

## Data Availability

The data that support the findings of this study are openly available in Zenodo at https://doi.org/10.5281/zenodo.6993871, reference number 6993871.
