# Supplementary material for: Graphene at Liquid Copper Catalysts: Atomic‐Scale Agreement of Experimental and First‐Principles Adsorption Height
Source: Adv Sci (Weinh). 2022 Nov 9;9(36):2204684. doi: 10.1002/advs.202204684 (PMC9798965; doi:10.1002/advs.202204684)
Supplement: Supplementary file 1 — Supporting Information [file ADVS-9-2204684-s001.pdf]

ADVANCED SCIENCE
Open Access

Supporting Information

for *Adv. Sci.*, DOI 10.1002/advs.202204684

Graphene at Liquid Copper Catalysts: Atomic-Scale Agreement of Experimental and First-Principles Adsorption Height

Hao Gao, Valentina Belova, Francesco La Porta, Juan Santiago Cingolani, Mie Andersen, Mehdi Saedi, Oleg V. Konovalov, Maciej Jankowski, Hendrik H. Heenen, Irene M. N. Groot, Gilles Renaud and Karsten Reuter*

Supporting Information of “Graphene at Liquid Copper Catalysts: Atomic-Scale Agreement of Experimental and First-Principles Adsorption Height”

Experimental details. The graphene was grown in a dedicated CVD reactor designed for *in situ* characterization compatible with optical microscopy, Raman spectroscopy, and X-ray diffraction and reflectivity techniques [1]. A puddle of liquid Cu was used as a catalyst. For this, a few round pieces of 50-µm-thick Cu foils with a diameter of ~15 mm (99.9976% purity, purchased from Advent Research Materials, The United Kingdom) placed on a tungsten disk were melted and annealed above the melting point (1357 K) in Ar(91%)/H_2(9%) atmosphere at a total pressure of 200 mbar (kept constant during the whole experiment) in the reactor prior to the Gr deposition. The Gr single layer was grown and characterized according to the protocol described in ref. .[2]. The deposition was carried out in a ‘pulse’ mode. In this mode, the methane precursor(7 sccm of 2% CH_4 mixture with p_{CH4} =2.36 and p_{H2} = 7.45 mbar, respectively, in Ar for 20 s) was accumulated for some time before opening the valve and introducing the precursor into the reactor. After opening the CH_4 valve, the methane flow was stabilized at 7 sccm, equivalent to p_{CH4}/p_{H2} = 0.0127 (with p_{CH4} = 0.22 and p_{H2} = 17.19 mbar, respectively, at a total pressure of 200 mbar), until the layer was complete. The growth monitoring in real time was done through a quartz window on top of the reactor, where an optical microscope operated in radiation mode combined with a digital camera for image recording was installed.

The *in situ* XRR measurements were performed at the ID10 beamline of the ESRF synchrotron (Grenoble, France). To ensure the reactor transparency for the X-ray radiation with the beam energy of 22 keV, an aluminum side wall of the reactor wall was replaced with a copy made of thin beryllium. The vertical beam size was 15 µm, while horizontal size was 26 µm. A two-dimensional MAXIPIX [3] detector was used. The XRR data from the curved liquid Cu surface were treated according to the method described recently in ref. [4] to eliminate the curvature's effect and extract the normalized reflectivity profiles. The resulting XRR curves were fitted using Refl1D software [5] to a slab model consisting of one (in case of bare Cu) or three (in case of Cu covered with Gr) layers: a Cu substrate, a void, and a C monolayer. The density of the liquid Cu, the C layer, and the thickness of the C layer were fixed to 7.99 g/cm^3, [6] 5.36 g/cm^3 (derived from the graphite density [7], and 1.42 Å (diameter of the carbon atom [8]), respectively. The fitting parameters were the roughness of the Cu and Gr layers together with the width of the void layer. The roughness of the bare liquid Cu, of 1.1±0.1 Å, was obtained from fitting the reflectivity measured on it prior to Gr growth. This value was applied as a starting parameter for the fit of the Gr on the Cu curve. As a result, the roughness values of the Cu and Gr layer were 1.0±0.1 and 1.1±0.1 Å, respectively, and the void value was 1.5±0.1 Å. Note that both parameters (the roughness and the void width) are independent of each other.

Density Functional Theory Calculations. All the first-principles calculations were performed by the full-potential, all-electron DFT package FHI-aims [9] using light default settings for the basis set and integration grids, the PBE [10] or rSCAN [11] exchange-correlation functional, and a dipole correction. Dispersion corrections were included by either the Tkatchenko-Scheffler (TS) method [12] or via the MBD scheme [13]. K-point sampling was done with a density exceeding 60/Å k-points. The slab model for Cu(111) comprised 4 atomic layers with a (1x1) surface unit-cell, which then hosted 2 C atoms of a slightly strained Gr sheet (Gr on Cu(111) in Table S1). The liquid Cu slab model has a comparable thickness. Electronic self-consistency converged energies to within 10^{-6} eV, with the exception of large Gr-Cu(l) configurations computed with rSCAN, where a looser criterion of 10^{-2} eV was used. Bulk fcc Cu and solid Cu(111) geometries were relaxed until residual forces fell below 1 meV/Å and 10 meV/Å, respectively.

Training of machine learning potentials The ML potential was trained based on the Moment Tensor Potential formalism [14] with hyperparameters of level=20 and a cut-off of 6 Å. The potential was trained against a dataset computed with different DFT approximations (see above). The training set includes multiple configurations corresponding to bulk and surface of the solid and liquid phases. Initial configurations were chosen from previous work [15] and then augmented by an iterative trial and error approach to improve the description of the interface in simulation cells tractable with DFT. The size of the final training and test set is 44 and 9, respectively. Multiple MTPs are trained by the same training set starting from different random seeds, and the final potential is selected based on both the training errors and the relaxed lattice parameters of fcc Cu and Gr. In addition, for every DFT approximation, the potential is retrained on a subset of the training set, which only contains bulk Cu and Cu surface (17 configurations). The effect of Cu-C training data on the Cu-Cu interactions in MTPs is eliminated in this way and the retrained potentials are only used for bulk Cu and Cu surface in Figure 7. The details of training and test configurations are summarized in Table S1 and all configurations are publicly accessible in Zenodo (https://doi.org/10.5281/zenodo.6993871).

Table S1. Details of training and test configurations.

Description	Formula	Number of training configurations	Number of test configurations
fcc Cu (with different lattice constants)	Cu_4	4	
free Gr (with different lattice constants)	C_2	3	1
liquid Cu	Cu_{108}	1	1
Gr on frozen liquid Cu slab (varying Gr height)	$C_{32}Cu_{64}$	16	2
Gr supercell	C_{32}	1	
liquid Cu slab	Cu_{64}	1	1
Cu(111)	Cu_4	1	
Gr on Cu(111)	C_2Cu_4	1	
Cu(111) (331 supercell, rattled)	Cu_{36}	5	1
fcc Cu (222 supercell, rattled)	Cu_{32}	5	1
Gr on Cu(111) (331 supercell, varying Gr height)	$C_{18}Cu_{36}$	5	1

graphene flake on liquid Cu slab [15]	$C_{54}Cu_{1589}$	1	1

Molecular Dynamics Simulations

The MD simulations are propagated for 1 ns with a time step of 1 fs. A Nose-Hoover thermostat [16] and a Parrinello-Rahman barostat [17] with damping parameters of 0.1 ps for temperature and 1 ps for pressure were employed to produce an NVT or NPT ensemble at various temperatures, respectively. To compute the adsorption height of Gr above liquid Cu, a cell composed by a liquid Cu slab (1589 atoms) and a Gr layer (364 atoms) is used. The simulation cell dimensions are adjusted to the 0K lattice constant for Gr. Since the liquid Cu slab can dynamically adjust its thickness and Gr has a small thermal expansion coefficient [18], this model system introduces minimal strain. For solid Cu(111), the simulation cell dimension matches the Cu lattice constant in order to avoid any strain in the metal slab. The Gr sheet is fitted into the simulation cell whereby an almost negligible strain of -0.1% is introduced in a cell that contains a solid Cu slab of 11664 atoms and a Gr layer of 3136 atoms. To evaluate the strain effect on the adsorption height, we build two further models for Gr-Cu(111), which contain 1680 atoms with +3.6% strain (1344 Cu atoms and 336 C atoms), and 11716 atoms with -0.5% strain (9216 Cu atoms and 2500 C atoms), respectively. All the solid Cu slabs have a thickness of 8 layers.

The structural data of these simulations were used to evaluate an averaged electron density profile along the surface normal (see Figure 3). To process the electron density profile, we smear a Gaussian function over the laterally-averaged atom density (see Figure S5). The area of the Gaussian corresponds to the number of electrons (29 for Cu and 6 for C), and the width σ is chosen similar to the experimentally determined roughness factors (σ =1.0 Å for Cu and σ =1.1 Å for C, see above). Specifically, we employed σ=0.9 Å for Cu and σ=1.1 Å for C, which led to the best match to the shape of the experimental profile. The small difference between experimental and theoretical width for Cu results from a fit via an error function in one case and a Gaussian in the other. We note that fitting the resulting electron density with an error function reproduces the choice of σ =1.0 Å for Cu in the experiment.

The inflection-point "gap" is determined as the distance between the average position of the C atoms and the inflection point of the Cu density profile (see Figure 2, 3 and S7). In order to determine the inflection point, we fit an error function to the electron density of Cu of which the inflection point and the half maximum coincide (see Figure S5b). This procedure is robust in comparison to numerically computing the second derivatives, which is prone to noise. We emphasize, that the σ for both the Gaussians and the error function only affects the electron density profile shape but not the determined Gr-Cu(l) gap if chosen reasonably large (see Figures S2 and S3). Furthermore, the averaged Gr-Cu "gap" is computed over MD trajectory segments of 0.1 ns over the last 0.9 ns and the uncertainties are derived from the standard deviation between these segments.

A 1 ns NPT MD trajectory of a liquid Cu bulk cell (864 atoms) at 1356 K (the experimental melting temperature of Cu) and one atmosphere of pressure provides the density of liquid Cu by every potential. For every potential, the NPT simulation repeats 3 times with different random seeds to get the mean value and the uncertainty of liquid density. The surface tension is evaluated from 1 ns MD simulations at 1370 K of a liquid Cu slab (1589 atoms) by this equation:

$\gamma = \frac{L}{2}\left\langle P_{ZZ} - \frac{1}{2}(P_{XX} + P_{YY})\right\rangle$ where $P_{\alpha\alpha}$ is the $\alpha\alpha$ element of the pressure tensor and L is the length of the simulation cell along the z-axis. [19]. The way to compute the mean value and the uncertainty of the surface tension is just similar to the "gap". All the bond angles inside the Gr layer in the last 0.9 ns are collected to obtain the average bond angle to coordinating C atoms for every C atom and the signed delta angle is defined as $\delta\theta = sign(d_z)(120 - \overline{\theta})$, where $\overline{\theta}$ is the average bond angle of a C atom and d_z is the z component of the displacement from the central atom to the centroid of its three neighbors. Here the sign of $\delta\theta$ depends on whether the central atom is beneath or above its neighbors relative to the surface normal. The cell for the adsorption gap and energy of Gr on Cu(111) at 0K contains 4 Cu atoms and 2 C atoms and the C-C bond length in Gr is adjusted according to the relaxed fcc Cu (same as DFT calculations). The average adsorption energy at high temperature is obtained by the trajectory of the last 0.9 ns from the 1 ns simulation of the free-standing Gr, the liquid/solid slab and Gr on the slab. We used the atomic simulation environment (ASE) and LAMMPS for data handling and to perform simulations [20, 21].

Comparison of the inflection-point and direct "gap"

To characterize the adsorption height of Gr on the solid Cu(111), we also compute the gap by a direct method. Here, the gap is determined as the distance between the (center of) the top surface atoms of the solid Cu slab and those of the Gr layer. Since the surface atoms are clearly defined for the solid slab, we can easily determine the average value and standard deviation of all the "direct" distances (see Figure S5) and sample them analog to the inflection-point "gap". This gap value corresponds to the experimentally determined distances for solid Cu [22, 23] (and other metals). An inflection-point "gap" on the solid slab is in contrast not reported to our knowledge. Therefore, a straightforward comparison cannot be easily done based on literature data. On the other hand, such a direct "gap" for the liquid case is vague since a peak position is not sharp (see Figure S5d). Fortunately, through our simulations we can provide a comparison of the different "gaps" which is justified by the excellent agreement of both, the inflection-point gap for Gr on liquid Cu (see main text) and the direct gap for Gr on Cu(111). We determine this latter as 3.287(±0.0001) Å at 300K , which compares well to the experimental values of as 3.34 ± 0.06 Å [22] and 3.0 ± 0.2 Å [23] measured via *ex situ* total-reflection high-energy positron diffraction (TRHEPD) and atomic force microscopy (AFM) at room temperature. The direct value and the inflection-point determined gap in our simulations differ consistently by $\delta D \approx$ 1 Å at the employed σ values used for the electron density. This δD roughly stays constant with temperature and ML potential used. We therefore use this value (1 Å) to freely translate between the two "gap" definitions.

Table S2. Root mean square error (RMSE) of three ML potentials trained with different DFT reference data.

	MTP@PBE+TS	MTP@PBE+MBD	MTP@rSCAN	MTP@PBE
Training RMSE of energies (meV/atom)	1.6	2.0	1.3	1.5

Training RMSE of forces (meV/ Å)	48	47	55	52
Test RMSE of energies (meV/atom)	6.3	3.6	6.3	3.0
Test RMSE of forces (meV/ Å)	85	83	96	96

Table S3. Energy drift (meV/atoms/ns) in simulations driven by MTP@PBE+MBD.

Cell Size/Strain	300K	1000K	1100K	1200K	1370K
$Cu_{9216}C_{2500}$/-0.5%	-0.008	-0.1	-0.06	9.5	--
$Cu_{11664}C_{3136}$/-0.1%	-0.02	0.1	0.007	5.3	--
$Cu_{1344}C_{336}$/3.6%	0.0007	-0.3	0.02	7.7	--
$Cu_{1589}C_{364}$/0, liquid	--	0.4	-0.5	-0.1	0.2

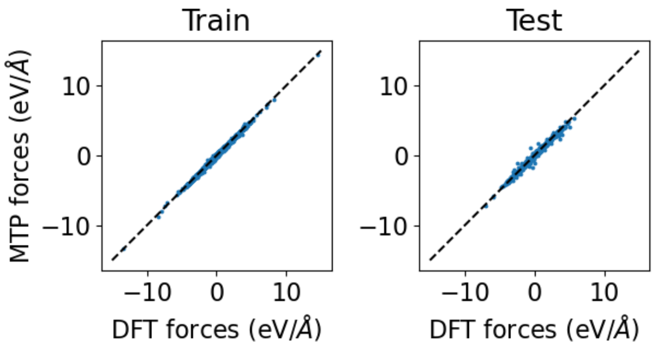

Figure S1 Comparison of force predicted by MTP (MTP@PBE+MBD) and DFT(PBE+MBD) on both training and test snapshots.

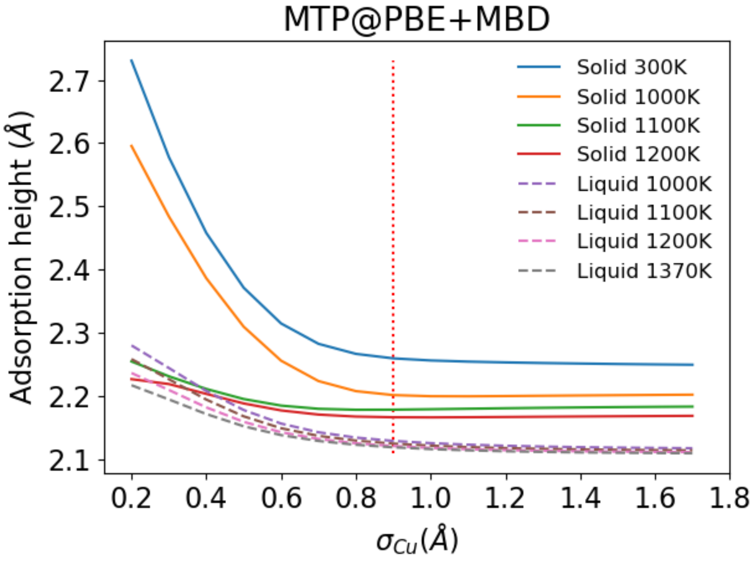

Figure S2 Inflection-point gap of Gr on liquid (Gr-Cu(l)) and solid Cu (Gr-Cu(111)) against the employed gaussian width of Cu for the electron density smearing for MTP@PBE+MBD. Here the strain of Gr on the solid slab is -0.1%.The red dotted vertical line indicates the Cu sigma employed in the main text.

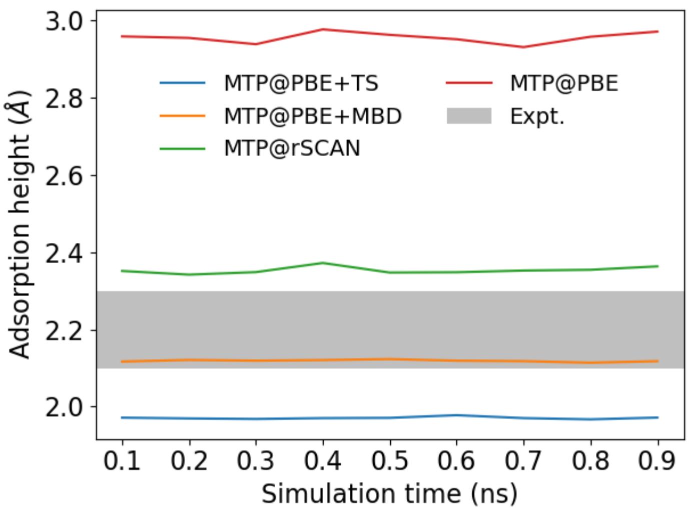

Figure S3 Inflection-point gap of gr on liquid Cu (Gr-Cu(l)) against simulation time for different MTP Potentials simulated at 1370 K. The total trajectory is 1ns long and the last trajectory of 0.9 ns are taken. The ”gap” is computed every 0.1 ns.

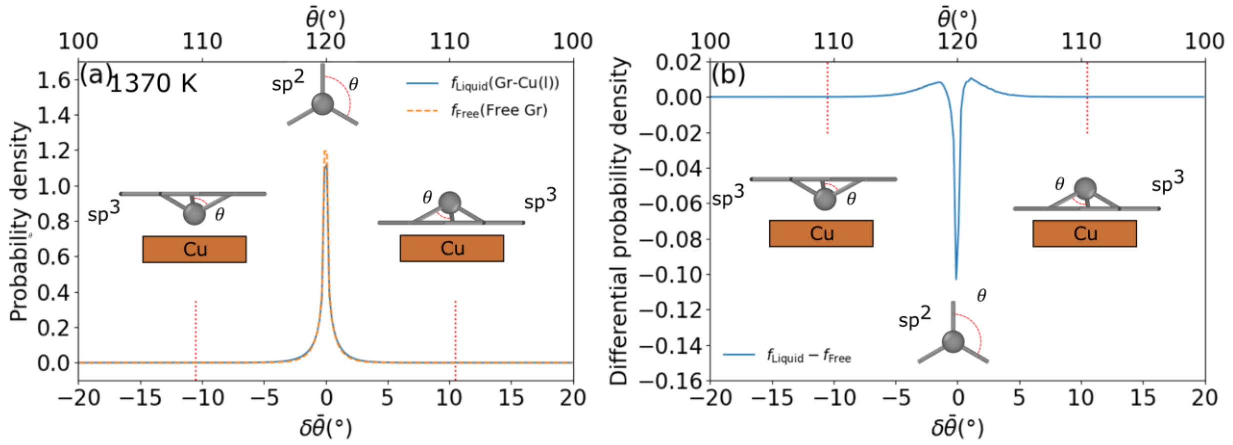

Figure S4 (a)Signed delta bond angles ($\delta\theta$) inside the Gr layer with and without liquid cu slab at 1370K. The red dotted line represent the typical bond angle in sp^3 hybridization. (b) The difference between the distributions of $\delta\theta$.

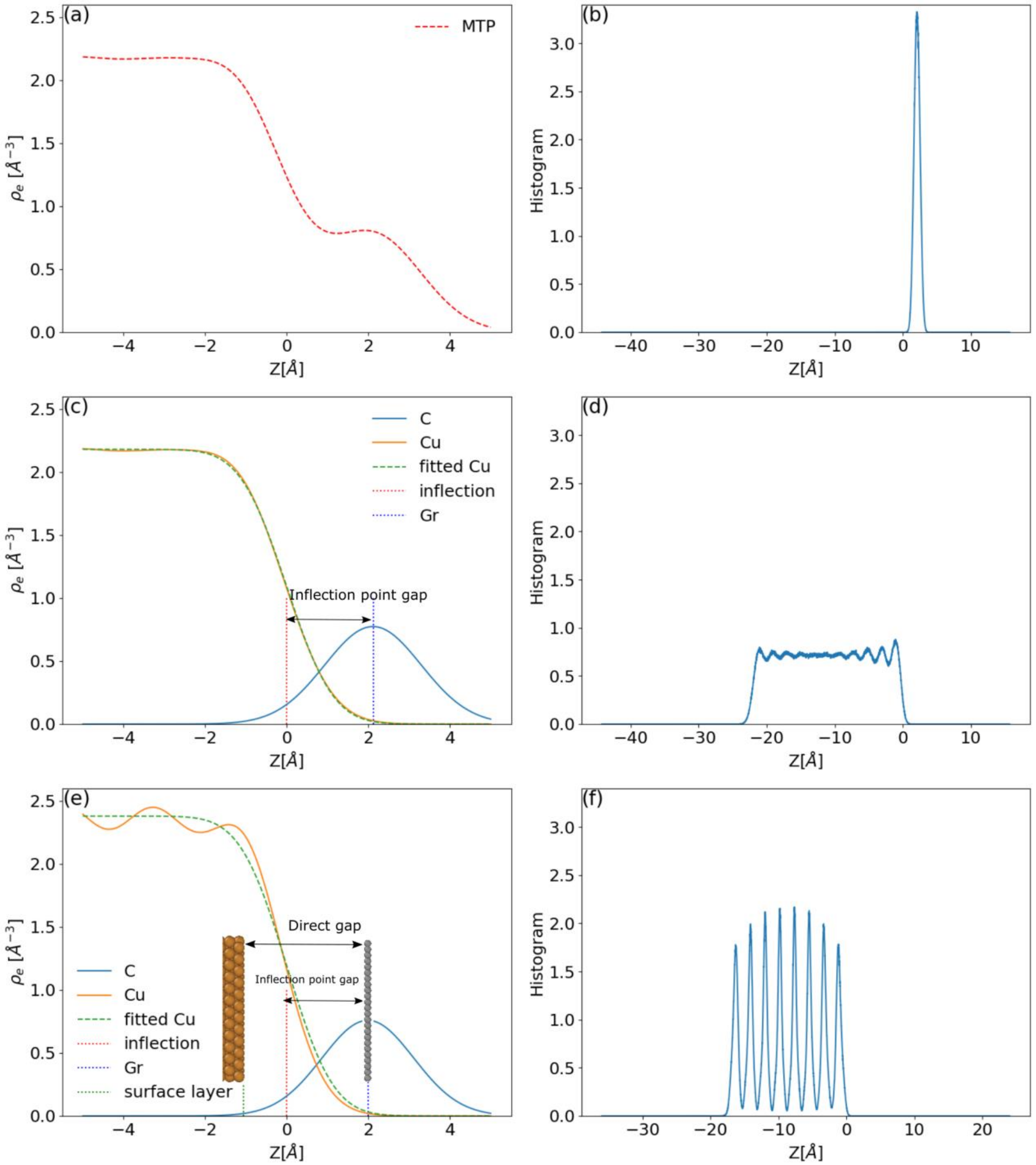

Figure S5 (a) Electron density profiles of Gr-Cu(l) at 1370 K computed by MTP@PBE+MBD-based MD. (b) Atomic density profile of C in Gr-Cu(l). (c) Species-resolved computed electron density profiles of Cu and C. The dashed green line is an error function fitted to the electron density profile of Cu, which is used to identify the inflection point. (d) Atomic density profile of Cu in Gr-Cu(l). (e) Species-resolved computed electron density profiles of Cu and C in Gr-Cu(111) at 1200K. The atomic densities present an average of the atom positions over the sampled MD trajectories and are the basis for the electron density which is computed using a gaussian smearing σ=0.9 for Cu and σ=1.1 for C (see details above). Vertical dotted lines denote the Cu density inflection point (Chosen as zero reference), the peak of the C Profile

(denoting the Gr position), and for the position of surface laypеr of Cu slab in (e). The “inflection-point gap” is derived as the distance between these two heights. The ”direct gap” is regarded as the surface layer and the Gr position. (f) Atomic density profile of Cu in Gr-Cu(111).

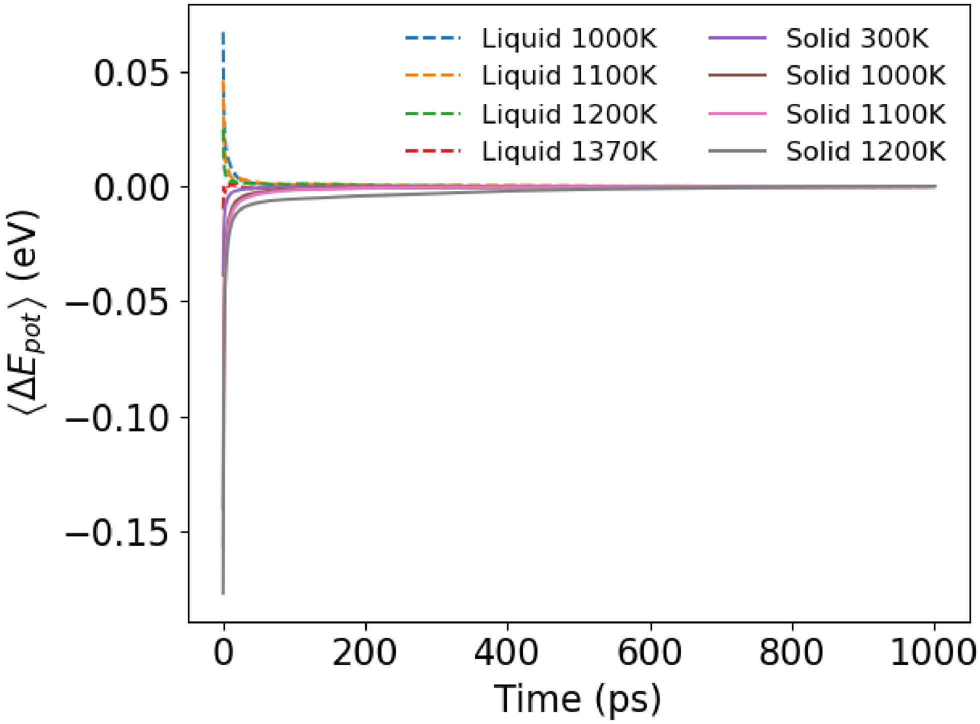

Figure S6 The cumulative average potential energy against simulation time for Gr on both solid and liquid Cu slab.

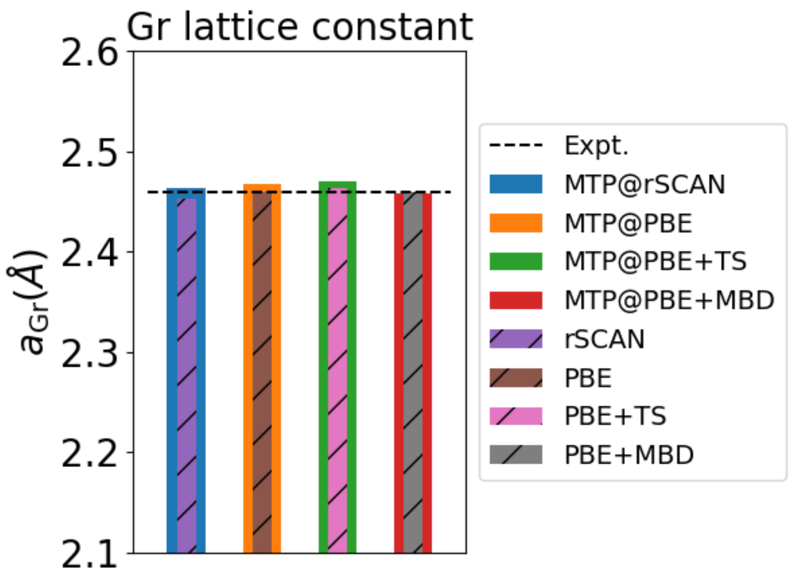

Figure S7 Lattice constant of Gr from the experiment [2] and calculations.

References

[1] M. Saedi, J. M. S. A. de Voogd, A. Manikas, C. Galiotis, M. Jankowski, G. Renaud, F. La Porta, O. Konovalov, G. J. C. van Baarle and I. M. N. Groot, "Development of a reactor for the in situ monitoring of 2D materials growth on liquid metal catalysts, using synchrotron x-ray scattering, Raman spectroscopy, and optical microscopy," *Rev. Sci. Instrum.,* vol. 91, p. 013907, 2020.

[2] M. Jankowski, M. Saedi, F. L. Porta, A. C. Manikas, C. Tsakonas, J. S. Cingolani, M. Andersen, M. d. Voogd, G. J. C. v. Baarle, K. Reuter, C. Galiotis, G. Renaud, O. V. Konovalov and I. Groot, "Real-Time Multiscale Monitoring and Tailoring of Graphene Growth on Liquid Copper," *ACS Nano,* vol. 15, no. 6, pp. 9638-9648, 2021.

[3] C. Fröjdh, H. Graafsma, H. E. Nilsson and C. Ponchut, "Characterization of a Pixellated CdTe Detector with Single-Photon Processing Readout," *Nuclear Instruments and Methods in Physics Research Section A: Accelerators, Spectrometers, Detectors and Associated Equipment ,* vol. 563, no. 1, p. 128–132, 2006.

[4] O. V. Konovalov, V. Belova, F. La Porta, M. Saedi, I. M. N. Groot, G. Renaud, I. Snigireva, A. Snigirev, M. Voevodina, C. Shen, A. Sartori, B. M. Murphy and M. Jankowski, "X-Ray Reflectivity from Curved Surfaces as Illustrated by a Graphene Layer on Molten Copper," *J. Synchrotron Rad.,* vol. 29, no. 3, p. 711–720, 2022.

[5] P. Kienzle, J. Krycka, N. Patel and I. Sahin, "Refl1D (Version 0.8.15) [Computer Software].," College Park, MD: University of Maryland., [Online]. Available:

https://refl1d.readthedocs.io/en/latest/getting_started/license.html. [Accessed 28 6 2022].

[6] J. A. Cahill and A. D. Kirshenbaum, "THE DENSITY OF LIQUID COPPER FROM ITS MELTING POINT (1356°K.) TO 2500°K. AND AN ESTIMATE OF ITS CRITICAL CONSTANTS," *J. Phys. Chem.,* vol. 66, no. 6, pp. 1080-1082, 1962.

[7] A. Savvatimskiy, "Measurements of the melting point of graphite and the properties of liquid carbon (a review for 1963–2003)," *Carbon,* vol. 43, no. 6, pp. 1115-1142, 2005.

[8] L. Pauling, The Nature of the Chemical Bond and the Structure of Molecules and Crystals: An Introduction to Modern Structural Chemistry, Cornell University Press, 1960.

[9] V. Blum, R. Gehrke, F. Hanke, P. Havu, V. Havu, X. Ren, K. Reuter and M. Scheffler, "Ab initio molecular simulations with numeric atom-centered orbitals," *Comput. Phys. Commun.,* vol. 180, no. 11, pp. 2175-2196, 2009.

[10] J. P. Perdew, K. Burke and M. Ernzerhof, "Generalized Gradient Approximation Made Simple," *Phys. Rev. Lett,* vol. 77, p. 3865, 1996.

[11] A. P. Bartók and J. R. Yates, "Regularized SCAN functional," *J. Chem. Phys.,* vol. 150, p. 161101, 2019.

[12] A. Tkatchenko and M. Scheffler, "Accurate Molecular Van Der Waals Interactions from Ground-State Electron Density and Free-Atom Reference Data," *Phys. Rev. Lett.,* vol. 102, p. 073005, 2009.

[13] J. Hermann and A. Tkatchenko, "Density Functional Model for van der Waals Interactions: Unifying Many-Body Atomic Approaches with Nonlocal Functionals," *Phys. Rev. Lett.,* vol. 124, p. 146401, 2020.

[14] I. S. Novikov, K. Gubaev, E. V. Podryabinkin and A. V. Shapeev, "The MLIP package: moment tensor potentials with MPI and active learning," *Mach. Learn.: Sci. Technol.,* vol. 2, p. 025002, 2021.

[15] J. S. Cingolani, M. Deimel, S. Köcher, C. Scheurer, K. Reuter and M. Andersen, "Interface between graphene and liquid Cu from molecular dynamics simulations," *J. Chem. Phys.,* vol. 153, p. 074702, 2020.

[16] M. E. Tuckerman, J. Alejandre, R. Lopez-Rendon, A. L. Jochim and G. J. Martyna, "A Liouville-operator derived measure-preserving integrator for molecular dynamics simulations in the isothermal-isobaric ensemble," *J. Phys. A: Math. Gen.,* vol. 39, p. 5629, 2006.

[17] M. Parrinello and R. A., "Polymorphic transitions in single crystals: A new molecular dynamics method," *Journal of Applied Physics,* vol. 52, no. 12, pp. 7182-7190, 1981.

[18] D. Yoon, Y.-W. Son and H. Cheong, "Negative Thermal Expansion Coefficient of Graphene Measured by Raman Spectroscopy," *Nano Lett.,* vol. 11, no. 8, pp. 3227-3231, 2011.

[19] J. Alejandre, D. J. Tildesley and G. A. Chapela, "Molecular dynamics simulation of the orthobaric

densities and surface tension of water," *J. Chem. Phys.,* vol. 102, pp. 4574-4583, 1995.

[20] A. H. Larsen, J. J. Mortensen, J. Blomqvist, I. E. Castelli, R. Christensen, M. Dujak, J. Friis, M. N. Groves, B. Hammer, C. Hargus, E. D. Hermes, P. C. Jennings and e. al, "The atomic simulation environment - a Python library for working with atoms," *J. Phys. Condens. Matter,* vol. 29, p. 273002, 2017.

[21] A. P. Thompson, H. M. Aktulga, R. Berger, D. S. Bolintineanu, W. M. Brown, P. S. Crozier, P. J. in 't Veld, A. Kohlmeyer, S. G. Moore, T. D. Nguyen, R. Shang, M. J. Stevens, J. Tranchida, C. Trott and S. J. Plimpton, "LAMMPS - a flexible simulation tool for particle-based materials modeling at the atomic, meso, and continuum scales," *Comput. Phys. Commun.,* vol. 271, p. 108171, 2022.

[22] Y. Fukaya, S. Entani, S. Sakai, I. Mochizuki, K. Wada, T. Hyodo and S.-i. Shamoto, "Spacing between graphene and metal substrates studied with total-reflection high-energy positron diffraction," *Carbon,* vol. 103, pp. 1-4, 2016.

[23] M. Schulzendorf, A. Hinaut, M. Kisiel, R. Jöhr, R. Pawlak, P. Restuccia, E. Meyer, M. C. Righi and T. Glatzel, "Altering the Properties of Graphene on Cu(111) by Intercalation of Potassium Bromide," *ACS Nano,* vol. 13, no. 5, pp. 5485-5492, 2019.
